# Reducing the burden of brain tumor surgery

**DOI:** 10.1007/s00701-020-04543-y

**Published:** 2020-09-01

**Authors:** Mark ter Laan, Suzanne Roelofs, Eddy M. M. Adang, Ronald H. M. A. Bartels

**Affiliations:** 1 Department of Neurosurgery, Radboud University Medical Center, Nijmegen, The Netherlands; 2 Department of Anesthesiology, Radboud University Medical Center, Nijmegen, The Netherlands; 3 Department for Health Evidence, Radboud University Medical Center, Nijmegen, The Netherlands

**Keywords:** Brain tumor, Neurosurgery, Health care costs, Post-operative care

## Abstract

**Background:**

Even though the need has been challenged, admitting patients to an intensive care or medium care unit (ICU/MCU) after adult supratentorial tumor craniotomy remains common practice. We have introduced a “no ICU, unless” policy for tumor craniotomy patients and evaluate costs, complications, and length of stay.

**Methods:**

A prospective cohort study was performed comparing patients that underwent tumor craniotomy for supratentorial tumors during 2 years after introduction of the new policy with the year before.

**Results:**

A reduction in ICU/MCU admittance from 88 to 23% of patients was found resulting in 13% cost reduction. Also, the new policy resulted in a 1.4-day shorter post-operative length of stay. Minor complications were reduced, while major complications remained the same. All major complications are reviewed.

**Conclusions:**

We show that routine post-operative ICU/MCU admittance after tumor craniotomy does not reduce complications, but actually interferes with recovery of our patients. Changing the paradigm results in earlier discharge and cost reduction.

**Electronic supplementary material:**

The online version of this article (10.1007/s00701-020-04543-y) contains supplementary material, which is available to authorized users.

## Introduction

Previously, the need for post-operative admittance to medium or intensive care units after craniotomy was challenged [1–4, 6–8]. The main reason for changing the post-operative regimen was the fact that patients on average reported quick recovery after tumor craniotomy, but complained about the burden of the stay at the ICU post-operatively. A retrospective analysis of our own complications showed a very low incidence, and therefore, we questioned the need for intensive post-operative monitoring. Also, a change in regimen could possibly reduce the burden on ICU planning (which has led to postponing surgery) and increase cost efficiency. After introduction of a “no-ICU-unless” protocol for our craniotomy patients we previously reported short-term results and found satisfied patients, comparable complication rates, and significant reduction of costs [7]. In this new report we present the results 2 years after introduction of this protocol was implemented. This allows for inclusion of more than twice the number of patients and detecting more significant differences.

## Materials and methods

For analysis only adults with elective open surgery for supratentorial lesions (extra-axial, intra-axial, skull base or cysts) were included. Purely calvarian bone tumors, pituitary tumors, and endoscopic procedures were excluded for analysis. Details of our post-operative regimen were reported previously [7]. In cohort A patients were routinely admitted to ICU or MCU post-operatively; in cohort B a “no-ICU-unless” policy was applied. This meant that indication for post-op intensive monitoring could be made by either the surgeon (criteria: length of surgery > 6 h, high expected blood loss, infratentorial location) or anesthesiologist (based on ASA score, co-morbidity and functional status).

Data were collected from April 1, 2016, until March 31, 2017, for cohort A (before the new protocol was implemented) and from April 1, 2017, until March 31, 2019, for cohort B. Length of stay and complications were extracted from hospital registries. Complications were graded according to Clavien-Dindo [5]. Costs were collected from the hospital billing system adhering to a third party payer perspective.

Differences in length of stay were analyzed by a general linear model including the following confounders: age, ASA score, procedure (intra-axial tumor, extra-axial tumors, skull base tumors, open biopsy, or open cyst fenestration), and use of intra-operative monitoring (IOM). Costs, usually skewed, were analyzed by a generalized linear model with a log link relating the conditional mean to confounder age, ASA score, procedure type, and IOM using a gamma distribution specifying the relationship between the variance and the mean. Statistical significance was assumed when *p* < 0.05. Statistical analyses were done using STATA 16.0 Statistical software (StataCorp LLC, Texas USA)

## Results

Cohort A consists of 107 patients; cohort B consists of 258 patients (Table 1). In cohort A 88% and in cohort B 23% of patients were admitted to ICU or MCU after surgery (data of cohort A differs a bit from our previous publication, because previously data were based on billing data and not all ICU stays were billed already). Age, sex, ASA score, and procedure type did not significantly differ between cohorts. Intra-operative monitoring was used more frequent in cohort A.

**Table 1:** Data of cohort A (April 1, 2016 untill March 31, 2017; routine ICU/MCU admittance post surgery, unless otherwise decided) and cohort B (April 1, 2017 until March 31, 2019; no routine ICU/MCU admittance post-surgery)

	“ICU, unless”	“No ICU unless”
*N*	107	258
Mean age	55.5	56.0
Admitted to ICU	88%	23%
No complication	38%	80%
Complication > CD2	6%	5%
Statistics-based on general linear model correcting for age, ASA score, IOM use, and procedure		
EMM total LOS	6.7d	5.2d*
EMM post-op LOS	5.4d	4.0d*
EMM total costs	€13207	€11515*

*EMM* estimated marginal means, *LOS* length of stay, *ASA* American association of anesthesiology, *IOM* intra-operative monitoring

*Significant difference, *p* < 0.01

### Length of stay

Analysis showed a difference in mean total length of stay (estimated marginal means) at the neurosurgical department (including 1 day prior to surgery, time at operating room, and ICU/MCU) of 1.5 days in favor of cohort B (*p* = 0.002). Further a difference in mean length of post-operative stay (estimated marginal means) of 1.4 days (*p* = 0.002) was seen.

### Complications

Complication rates differed insofar that Clavien–Dindo grade (CD) > 2 did not change between cohorts, whereas minor complications were reported much less in cohort B (as previously reported).

Twelve patients in cohort B had a complication CD > 2; six of those were initially admitted to the ICU/MCU (supplementary table). One had myocardial infarction on day 4 and died, one developed a severe pneumonia, and treatment was stopped because of poor oncological prognosis, and patient died, three had surgery for a complication (leakage of CSF, brain abcess, and perforated diverticulitis), and one patient needed ventilatory support after post-operative seizures. Of the six patients of cohort B with a severe complication that were not initially admitted to the ICU/MCU, two had surgery for an epidural hematoma (both at second post-op day), one had decompressive surgery because of venous infarction (second post-op day), one needed ventilatory support the evening after surgery because of drowsiness, and two had surgery for CSF leak or wound infection. Of those patients primarily assigned to the ward, only the patient that needed ventilatory support and the one with decompressive surgery had to be admitted to the ICU.

### Costs

Costs remained statistically significantly lower with a 13% reduction since introduction of this protocol (*p* = 0.001) with marginal cost per admission of €13207—and €11515—for respectively cohorts A and B. This means savings of €1700 on average per patient in the Dutch healthcare system, which is relevant from a costs perspective. The days admitted on ICU are responsible for about 75% of the difference in total cost (*p* < 0.001); meaning, this is the most important driver of the total cost difference between cohorts A and B. All the other cost sub-components (OR costs, costs associated with stay at ward, laboratory cost, imaging costs, and outpatient costs) together make up the other 25% of the total cost difference between both cohorts.

## Discussion

Our 2-year follow-up analysis substantiated the previous findings of reduced costs without decreasing safety when patients are not routinely admitted to a ICU or MCU after supratentorial tumor craniotomy. Furthermore, we showed that the reduced stay at the ICU or MCU is not replaced by an increased stay at the neurosurgical ward, but indeed leads to sooner discharge. This suggests that the new protocol creates sustainable value and that admittance of patients to a MCU or ICU after regular intracranial tumor surgery should be avoided, since this routine interferes with recovery of our patients.

Minor complications were significantly reduced after introduction of the new regimen. Possibly a reduction was achieved because of the shorter ICU/MCU stay: intravenous infusions are less frequent, catheters are discontinued sooner, and patients mobilize quicker at the ward. On the other hand, more intensive monitoring at the ICU/MCU units can lead to more detection of minor complications like hypertension and electrolyte problems.

We have described in detail the cases with severe complications. Half of the severe complications after introduction of our new regimen occurred in patients not initially admitted to an ICU or MCU. None of these would have been prevented by initial admission to the ICU or MCU department, only the patient that needed ventilatory support would have been saved a transfer, but close monitoring at the ward resulted in a timely diagnosis.

A limitation of our study is the fact that our sample size in not large enough to detect differences in occurrence of major complications, due to their low incidence. On the other hand, this means that the number of patients needed to admit to the ICU in order to prevent one complication is very high. Another limitation is de possibility of registration bias. Data for cohort A were collected retrospectively and data for cohort B prospectively, possibly resulting in registration of a higher incidence of complications in cohort B. This was not seen in our data. Finally, financial support by an insurance company might lead to a bias in favor of cutting costs. Study design, analysis, data management, interpretation, and composing of the manuscript have been completely independent.

One could argue our criteria for ICU/MCU admission are not strict nor specific enough. Possibly further study could identify better criteria for patient selection, but based on previous reports and our own results we propose to adopt our pragmatic approach of a “no-ICU-unless” policy to replace a “ICU-unless” policy. It is of utmost importance to identify those patients that do need ICU care and to facilitate monitoring at the ward. In short this means that patients who need to have a supratentorial craniotomy were high blood loss or long (> 6 h) surgery is anticipated by the surgeon or those that need extra monitoring based on ASA score, co-morbidity, or functional status do need post-op intensive care monitoring. We do recommend involving nursing staff in changing peri-operative regimen and increasing check-ups and monitoring possibilities on the ward for the first 6 h in order to facilitate such regimen.

## Electronic supplementary material

ESM 1 (DOCX 17 kb)
