# Supplementary material for: Reducing the burden of brain tumor surgery
Source: Acta Neurochir (Wien). 2020 Sep 1;163(7):1879–82. doi: 10.1007/s00701-020-04543-y (PMC8195912; doi:10.1007/s00701-020-04543-y)
Supplement: Supplementary file 1 — (DOCX 17 kb) [file 701_2020_4543_MOESM1_ESM.docx]

| Cohort | Sex | Age | ASA  score | Comorbidity | Tumour | Procedure | Post-op  ICU | Highest  CD | Remarks |
| --- | --- | --- | --- | --- | --- | --- | --- | --- | --- |
| A | M | 64 | 2 | Astma, mental retardation | Meningeoma | Extra Axial | Y | 4 | Venous infarction and epilepsy |
| A | F | 80 | 2 | Artrosis, cataract | Meningeoma | Extra Axial | Y | 4 | Post-op ventricular fibrillation and resuscitation |
| A | M | 59 | 2 | Esthesioneuroblastoma | Recurrent esthesioneuroblastoma | Extra Axial | Y | 4 | Returned to ICU because of ARDS and CVA |
| A | M | 48 | 3 | DM, astma, CVA, oligodendroglioma | Recurrent oligodendroglioma | Intra Axial | Y | 3 | Infected boneflap had to be removed |
| A | M | 66 | 3 | OSAS, cholecystitis, PE, viral encephalitis | Meningeoma | Skull Base | Y | 3 | Re-admitted for secondary hydrocephalus |
| A | M | 57 | 2 | Epilepsy | Diffuse astrocytoma | Intra Axial | Y | 3 | Surgery for woundinfection |
| B | M | 73 | 2 | DM, artrosis | Meningeoma | Extra Axial | Y | 5 | Myocradial infarction at day 4 in referring hospital |
| B | M | 57 | 3 | OSAS, HT, esophageal carcinoma, PE | Cerebral metastasis | Intra Axial | Y | 5 | PE and severe pneumonia, patiënt wished no further treatment |
| B | F | 33 | 4 | NF II, muscular dystrophia, | Meningeoma | Skull Base | Y | 4 | Intubated because of seizure |
| B | F | 62 | 2 | HT, hypercholesterolaemia, glaucoma | Meningeoma | Extra axial | Y | 3 | Surgery for perforated diverticulitis, atrial fibrillation, PE |
| B | M | 28 | 2 | None | Malignant meningeoma | Skull Base | Y | 3 | Repeat surgery because of CSF leakage |
| B | M | 64 | 2 | OSAS, CVA | Inflammatory disease | Open biopsy | Y | 3 | Repeat surgery because of abcess |
| B | M | 60 | 2 | CVA, HT | Oligodendroglioma | Intra Axial | N | 4 | Surgery for epidural hematoma at day 2; returned to ward post-op |
| B | F | 72 | 3 | Cataract | Primary cerebral lymphoma | Open biopsy | N | 4 | Opioid induced respiratory insufficiency, secondary ICU admittance |
| B | F | 45 | 2 | HT, PTSD | Meningeoma | Extra axial | N | 4 | Venous infarction that needed decompression at day 2 |
| B | M | 69 | 3 | Cataract, coloncarcinoma, PE | Craniofaryngeoma | Intra Axial | N | 3 | Repeat surgery because of CSF leakage, diabetes insipidus, PE |
| B | F | 63 | 2 | HT, aortic valve insufficiency, depression | Meningeoma | Extra axial | N | 3 | Repeat surgery because of woundinfection |
| B | M | 27 | 2 | None | Glioblastoma | Intra Axial | N | 4 | Surgery for epidural hematoma at day 2; returned to ward post-op |

Supplementary table 1: characteristics of all patients with complication > CD 2.

CVA = cerebrovascular accident, DM = diabetes mellitus, HT = hypertension, NF = neurofibromatosis, OSAS = obstructive sleep apnea syndrome, PE = pulmonary embolia, PTSD = post-traumatic stress disorder
